# Evaluation of Wear of Disc Brake Friction Linings and the Variability of the Friction Coefficient on the Basis of Vibroacoustic Signals

**DOI:** 10.3390/s21175927

**Published:** 2021-09-03

**Authors:** Wojciech Sawczuk, Dariusz Ulbrich, Jakub Kowalczyk, Agnieszka Merkisz-Guranowska

**Affiliations:** 1 Institute of Transport, Faculty of Transport and Civil Engineering, Poznan University of Technology, 60-965 Poznan, Poland; agnieszka.merkisz-guranowska@put.poznan.pl; 2 Institute of Machines and Motor Vehicles, Faculty of Transport and Civil Engineering, Poznan University of Technology, 60-965 Poznan, Poland; dariusz.ulbrich@put.poznan.pl (D.U.); jakub.kowalczyk@put.poznan.pl (J.K.)

**Keywords:** vibration, friction lining, friction coefficient, regression model

## Abstract

The article presents the results of friction and vibroacoustic tests of a railway disc brake carried out on a brake stand. The vibration signal generated by the friction linings provides information on their wear and offers evaluation of the braking process, i.e., changes in the average friction coefficient. The algorithm presents simple regression linear and non-linear models for the thickness of the friction linings and the average coefficient of friction based on the effective value of vibration acceleration. The vibration acceleration signals were analyzed in the amplitude and frequency domains. In both cases, satisfactory values of the dynamics of changes above 6 dB were obtained. In the case of spectral analysis using a mid-band filter, more accurate models of the friction lining thickness and the average coefficient of friction were obtained. However, the spectral analysis does not allow the estimation of the lining thickness and the friction coefficient at low braking speeds, i.e., 50 and 80 km/h. The analysis of amplitudes leads to the determination of models in the entire braking speed range from 50 to 200 km/h, despite the lower accuracy compared to the model, based on the spectral analysis. The vibroacoustic literature presents methods of diagnosis of the wear of various machine elements such as bearings or friction linings, based on amplitude or frequency analysis of vibrations. These signal analysis methods have their limitations with regard to their scope of use and the accuracy of diagnosis. There are no cases of simultaneous use of different methods of analysis. This article presents the simultaneous application of the amplitude and frequency methods in the analysis of vibroacoustic signals generated by brake linings. Moreover, algorithms for assessing the wear of friction linings and the average coefficient of friction were presented. The algorithm enables determination of the time at which the friction linings should be replaced with new ones. The final algorithm analyzes the vibration acceleration signals using both amplitude analysis for low braking speeds, as well as spectral analysis for medium and high braking speeds.

## 1. Introduction

Changes in the construction of disc brakes are related to the increase in train speed. The braking system absorbs and dissipates the vehicle’s energy to the environment as heat during the braking process. The unfavorable processes during the braking of rail vehicles include vibroacoustic processes in the brake system (vibrations, noise), thermal processes occurring in the friction pair, and wear of the brake components. The above processes affect the deterioration of the acoustic environment, especially the comfort of passengers and the operating costs of rail vehicles. Vibroacoustic phenomena emitted by brake systems are considered very undesirable. The problem of vibrations in the brakes of railway and car vehicles has been recognised since the 1930s [1,2].

Vibroacoustic processes generated by braking systems are analyzed in three groups. The first group covers works on noise during the braking process; there are articles that attempt to recognize and identify the cause of noise [3,4,5,6,7,8], while other scientists are trying to model noise depending on the geometric features of the brake system, as well as the elements of the brake friction pair. In the second, less numerous group of publications on vibroacoustic processes in braking systems, there are works related to the use of vibrations of the braking system to assess the wear condition of brake friction elements [9,10]. In [11], the authors presented the possibility of using resonant vibrations in the assessment of wear of friction linings. These tests were preceded by a modal analysis of the braking system components in order to determine the resonance frequencies of these components. Then, the wear of the friction linings was assessed at these frequencies. The paper [12] presents the analysis of vibration acceleration signals in the frequency domain using band filters, from which the effective value of vibration accelerations was determined. The work [13] contains the results of the analysis in the amplitudes domain using known measures. For the purpose of the diagnosis of friction lining wear, the article analyzes such measures as the effective value of vibration acceleration (A_RMS_), the average value (A_AVERAGE_), the root value (A_SQUARE_), and the peak value of the vibration acceleration (A_PEAK_). From this analysis, two point measures were selected, i.e., A_RMS_ and A_AVERAGE_, which, due to the smallest relative error in diagnosis, are best suited for the price and estimation of the wear value of rail brake linings. The least numerous group are those dealing with the use of vibrations of the braking system also in the assessment of the braking process [14,15]. The work [16] is the first attempt to present the dependence of the friction coefficient on vibrations of friction linings, which in the future may be used in the evaluation of the braking process. It was demonstrated only in the example of three frequency characteristics from braking from the speeds of 120, 160 and 200 km/h.

**Figure 1:**
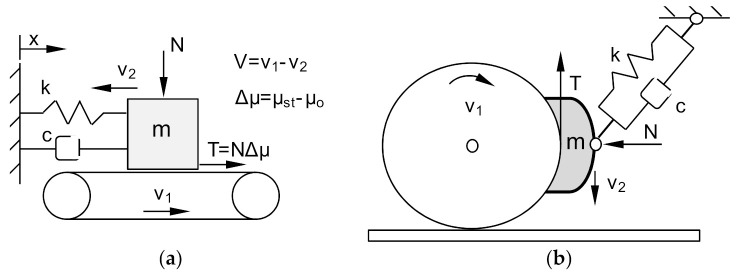
Brake system models: (**a**) Model of an elastic friction system on the example of a conveyor belt (stick-slip phenomenon), k—spring stiffness, c—viscous damping coefficient, m—block mass, N—pressure force on the conveyor belt, T—friction, V—relative speed, v_1_—conveyor belt speed, v_2_—pad speed, µ_st_—static friction coefficient, µ_o_—kinetic friction coefficient, x—displacement; (**b**) Model of the actual brake system 1Bg in the railway block brake.

Most of the models describing the vibrations of the braking system are based on the assumption that the increase in vibrations, as a phenomenon strongly dependent on many variables, is largely influenced by the variability of the friction coefficient between the brake lining and the brake disc throughout the braking process. Additionally, the susceptibility of the braking system, particularly brake linings, contributes to increase and to reduce the vibrations generated by the braking system. The first models assumed that the self-excited vibrations of the brake were related to a decrease in the friction coefficient and an increase in the sliding velocity (Figure 1). This is true for many friction materials, but for a limited range of speed changes, which was already described in the works [1,7].

In 1961, Fosberry and Holubecki [17] found that in brake systems, vibrations caused by the cooperation of the pad-disc friction pair have a static friction coefficient μ_st_ higher than the kinetic friction coefficient μ_k_, or the kinetic friction coefficient decreases with increasing velocity v_2_. Similar conclusions were contained in the work of Sinclair [6] from 1955 and Earles [18]. Other researchers, such as Mills, Bowden and Leben [19] conducted research on elastic friction systems, comparing them to stick-slip motion. The main conclusion from this research was that the vibroacoustic events have not been fully established. The most likely explanation of these phenomena is stick-slip motion occurring in the frictional coupling. The energy source of this motion is the change of the friction coefficient as a function of velocity [4]. However, the stick-slip model does not take into account the effect of system damping.

**Figure 2:**
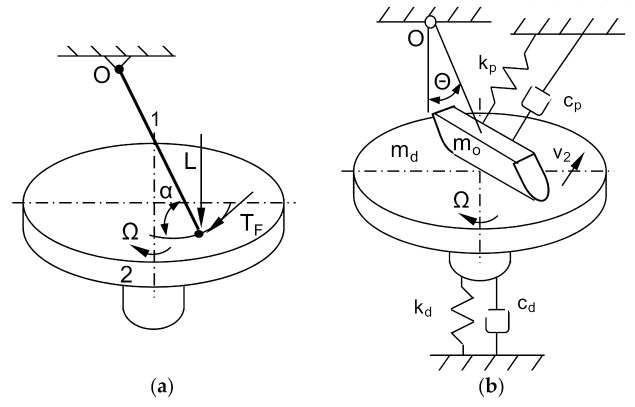
Models of brake systems: (**a**) Model of the sprag-slip phenomenon according to Spurr, 1—stiff rod rotating at point O, 2—stiff rotating disc; (**b**) Model of the friction disc-slider system on the Jarvis and Earles angle cantilever, α—slope angle of cantilever in the horizontal plane, Z—bracket width in m, R—bracket rotation radius in m, Ω—plate rotation speed in rpm, θ—cantilever slope angle in the vertical plane, v_2_—slider linear speed in m/s, m_o_—weight of the cantilever in kg, m_d_—weight of the panel in kg, c_p_—cantilever damping, k_p_—cantilever stiffness, c_d_—disc damping, k_d_—disc stiffness.

Spurr in [20] proposed the term sprag-slip to describe vibroacoustic phenomena in brake systems. According to this term, the vibroacoustic phenomena generated by brake systems result from the contact of the friction material with the brake disc. The friction force in such a system can be much greater than the force in a perfectly rigid system. In the real braking system, due to elastic deformations and displacement of elements, there is a cyclical momentary decrease and increase in the value of the friction force. The model of the sprag-slip phenomenon of a stiff bar fixed at point O and loaded with an external force L acting on the other end of the bar at the point of contact with the stiff displacing surface is shown in Figure 2a.

If *T_F_* = *_μk_N*, the equilibrium of the system will be maintained on the basis of Equation (1) presented in [17]:

(1) $$T_{F}=\frac{\mu_{k}L}{1-\mu_{k}\tan\left(\alpha \right)}\;$$

If the angle of inclination of the stiff rod *α* tends to the value of tan^−1^(1/*μ_k_*), then the friction force *T_F_* will approach infinity. Spurr referred this particular case as spragging. This model was then refined by Jarvis and Earles, as shown in Figure 2b [7].

The brake instability condition was defined by the relationship [7]:

(2) $$\frac{1}{2}\left(\mu_{k}-\tan\theta \sin\right)2\theta >\frac{C_{p}}{C_{d}}\;$$

It was the first attempt of theoretical presentation of the sprag-slip phenomenon. A rotating plate with an attached cantilever was used to explain the vibroacoustic phenomena. Later models based on the considerations of Jarvis, Mills and Earles were shown. These models were more complicated, with more and more degrees of freedom and several friction models. Another model was proposed by North, and then by Millner. It was a model of the binary flutter, which is more similar to a disc brake [7,10]. The mechanism of vibrations in this case is similar to vibrations during the flapping of wings in airplanes, i.e., flutter. The disc brake track has been replaced with a rigid straight beam with two degrees of freedom. Crolla and Lang found that this and other models do not fully reflect the actual brake construction. However, thanks to their research, it is possible to obtain a qualitative hint at the design process. In addition, solutions that eliminate some classes of brake vibrations and the emitted noise can be designed [7].

Lang and Smales in [3] discerned two types of vibroacoustic events coming from braking systems. This distinction also applies today. The phenomena occur at low frequencies—from 1 to 5 kHz and at high frequencies, above 5 kHz. The Lang and Smales model in the low frequency range allows for the treatment of the friction lining as a rigid body. However, at high frequencies, the deformation of the friction elements should be additionally taken into account. The assumption of a rigid body as a friction material was also taken into consideration by Brooks [10] and Milner [21], as well as Rudolph and Popp [5,22]. In addition, the susceptibility of the brake system components, in particular the brake friction linings, increases the wear of the friction material (its emission to the environment—a highly unfavorable phenomenon) and decreases vibrations generated by the brake system (a favorable phenomenon). The collected results from the operation of the braking system allow for its reliability analysis [23,24].

## 2. Materials and Methods

**Figure 3:**
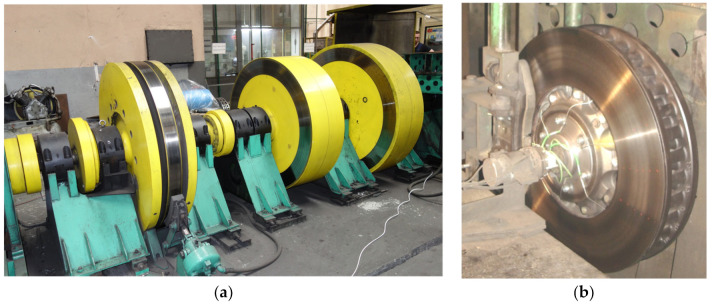
Stand for testing railway disc brakes: (**a**) driving part of the brake stand with rotating masses, (**b**) brake disc type 610 × 110 mm mounted on the brake stand.

Determination of selected frictional and vibroacoustic characteristics, depending on the parameters of the braking process, was carried out. During the tests, the input parameters were intentionally and in a specific way changed, i.e., friction linings of different thickness, braking speed, and their influence on the changes in the output parameters were observed. The initial parameters were the instantaneous vibration accelerations recorded on the mounts with friction linings. In particular, attention was paid to the influence of the condition of the braking system understood as the wear of friction linings (linings of different thickness) on the frictional characteristics (average coefficient of friction as a function of speed) and vibroacoustic characteristics (effective value and average of vibration acceleration determined during braking from a given speed on the lining at a specific thickness). The tests were carried out on the inertia brake stand, shown in Figure 3. The test stand allows for testing the rail block and disc brake, reflecting the actual conditions that occur during braking of the rail vehicle.

The tests were carried out on a ventilated brake disc with dimensions of Ø610 × 110 mm, made of gray cast iron. The brake disc was prepared for tests in accordance with the EN 14535-1:2005 standard [25]. The linings (Frimatrail Frenoplast S.A., Wołomin, Poland), in accordance with the manufacturer’s procedure and the requirements contained in [26], were made of thermohardening resin, synthetic elastomer, metal and organic fibers, and friction modifiers.

**Figure 4:**
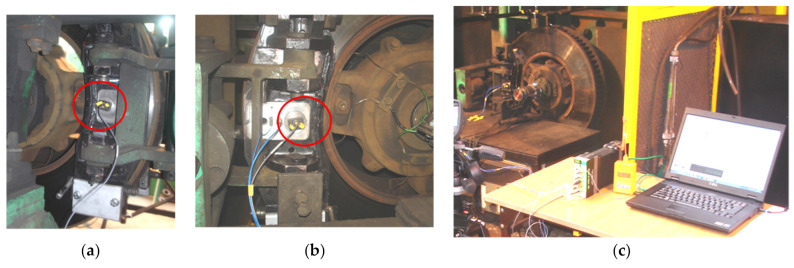
The tested brake disc mounted on the brake stand for testing railway disc brakes: (**a**) view of the left friction pair—brake disc with lining and attached vibration transducer, (**b**) view of the right brake holder and attached vibration transducer, (**c**) view of the stand with vibration measurement equipment.

Three sets of linings were used for the stand tests. One new set of linings (4 pieces) had a thickness of G_1_ = 35 mm and two sets used up to a thickness of G_2_ = 25 mm and G_3_ = 15 mm. The friction lining weights were, respectively, m_G1_ = 1.75 kg (new lining), m_G2_ = 1.45 kg (lining worn to a thickness of 25 mm), and m_G3_ = 1.02 kg (lining worn to a thickness of 15 mm). The vibroacoustic tests were carried out in parallel with the frictional (tribological) tests. One vibration transducer was attached to the brake system (right and left) as is shown in Figure 4a,b. The vibration transducers were bolted to the brake carrier plates to increase the vibration measurement range. The input quantities were the simulated braking initiation speed v_o_, the brake pad pressure on the brake disc N, the brake mass M and the friction lining thickness G. The output signals were the instantaneous tangential force F_t_ related to the braking radius r_h_, the instantaneous pressure force on the brake disc F_b_, as well as the instantaneous acceleration value of vibrations a. Thanks to this, it was possible to observe the influence of changing the input parameters on the obtained output signal.

Figure 4c shows the brake stand with the measuring equipment. The HBM type sensor was used to measure the force and the vibrations were measured by the B&K 4504A transducers.

Vibration transducers should be located close to the place generating the vibroacoustic signal (signal from the brake friction pair) and in a place easily accessible for the measurement. The vibration accelerations were measured in the perpendicular direction to the surface of the brake disc, based on the experience of other researchers presented in the works [27].

**Figure 5:**
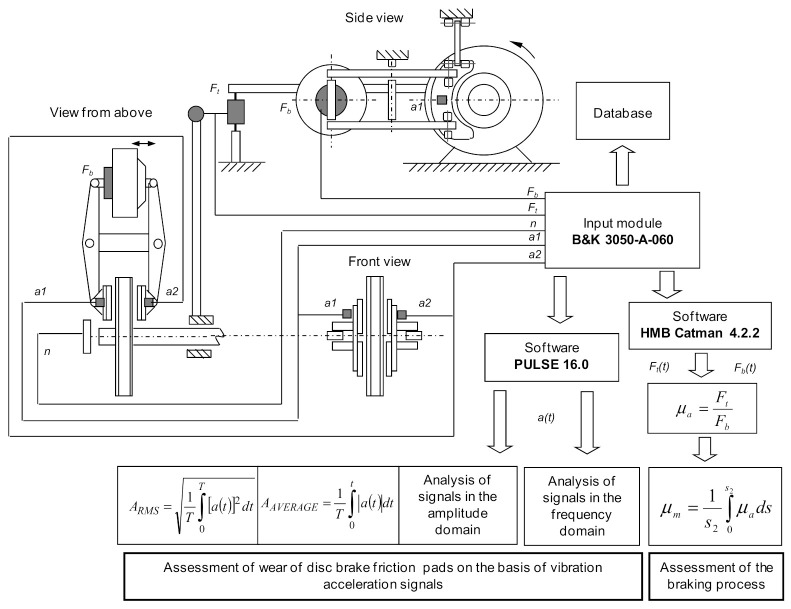
Diagram of the measuring system used during vibroacoustic stand tests.

Figure 5 shows a diagram of the measuring system used in the brake stand, extended with the measurement of vibration acceleration.

The linear frequency response of the converters was 13 kHz. During the diagnostic tests, signals in the 0.1 Hz to 9 kHz band were recorded. The sampling frequency was set to 32 kHz. This means that the band analyzed according to the Nyquist relationship was 16 kHz.

## 3. Results

**Figure 6:**
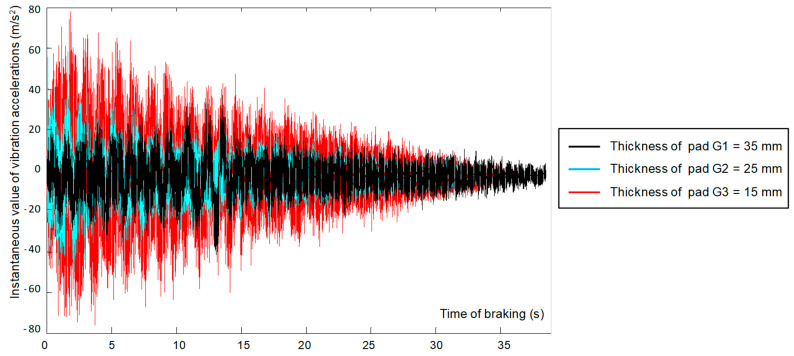
Signal of vibration accelerations registered on lining cladding for different thicknesses of linings during braking to stop (speed at beginning of braking *v* = 120 km/h).

The obtained signals of vibration acceleration generated by the braking system during braking were analyzed in the time domain. Figure 6 shows the changes of the instantaneous vibration accelerations recorded during slope braking. Then, the signals for each of the claddings were analyzed in the domain of amplitudes and frequency.

Based on the changes presented in Figure 6, it can be concluded that the wear of the friction material affects the increase in the amplitude of vibration acceleration from the holder with friction linings. Therefore, there is a dependence of the vibrations measured on the brake holder on the condition of the brake system (lining wear). Determining the dependence of the average coefficient of friction on the vibrations generated by the braking system required measurements for all considered braking speeds and recording of the instantaneous vibration acceleration of the brake holders with linings (for both sides of the brake disc).

For the analysis of vibration signals in the amplitude domain, measures are often used, which characterize a vibration process with one value [28]. Then, especially in vibroacoustic diagnostics (WA), it is possible to determine changes in the WA signal resulting from a change in the technical condition of the tested object. There are many publications in the literature presenting the use of vibroacoustic diagnostics (in some cases in combination with thermal imaging diagnostics) in automotive, railroad or air vehicles [19,29,30,31,32,33,34,35,36].

The measures used in vibroacoustic diagnostics are divided into dimensional and non-dimensional points [28]. The measures used to diagnose the wear of the friction linings of the railway disc brake were as follows:

- average amplitude, presented with the relationship:

(3) $$A_{\mathrm{AVERAGE}}=\frac{1}{T}\int_{0}^{T}\left|s\left(t\right)\right|dt$$

where: *T*—averaging time, *s*(*t*)—instantaneous value of the amplitude of displacements, velocities or accelerations of vibrations;

- effective amplitude, described by the equation:

(4) $$A_{\mathrm{RMS}}=\sqrt{\frac{1}{T}\int_{0}^{T}{\left[s\left(t\right)\right]}^{2}dt}$$

In the vibroacoustic tests it was observed that for the measurement of the vibration accelerations on both sides of the brake disc during braking, different values of the signals were recorded. The vibrations measured on the brake holder from the side of the brake cylinder are characterized by a higher value of vibration accelerations than those measured from the side of the piston rod brake. The various values were obtained, despite the fact that the lever mechanism is characterized by a symmetrical construction of the right and left sides. A detailed explanation of this phenomenon is described in [37]. For further analysis, the results measured on the holder from the side of the brake cylinder were used. Before calculating measures from vibration acceleration signals, a time selection was made in the MATLAB program. As a result of this analysis, the part related only to the braking process was separated from the entire recorded signal in order to obtain the required dynamics of changes, important for diagnostic purposes. The dependence of the friction lining thickness on the selected measures was determined by the dynamics of changes for a given parameter, which is shown in the following relationship [38]:

(5) $$D_{1\left(2\right)}=20log_{10}\left(\frac{A_{2}}{A_{1}}\right)$$

where: *A*_1_—value of the measure determined for the *G*_1_ cladding, *A*_2_—value of the measure determined for the *G*_3_ or *G*_2_ cladding.

In terms of frictional characteristics, the tangential force F_t_ and the pressure force on the brake disc F_b_ were measured on the brake stand in order to determine the instantaneous coefficient of friction *μ_a_*. Then, the average coefficient of friction was calculated according to the relationship where s is the measured braking distance [26].

(6) $$\mu_{m}=\frac{1}{s_{2}}\int_{0}^{s_{2}}\mu \times ds$$

**Figure 7:**
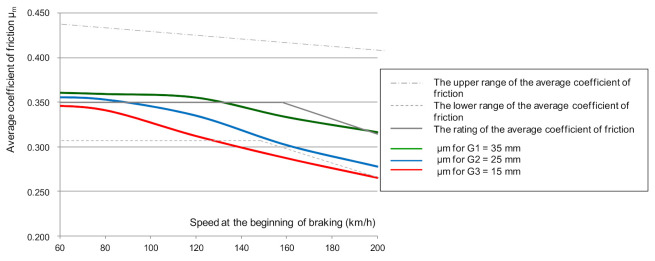
Dependence of the average coefficient of friction *μ_m_* on the set braking start speed at N = 25 kN and M = 5.7 t at different friction lining thicknesses.

Figure 7 shows the dependence of the average coefficient of friction *μ_m_* depending on the braking start speed, with the brake pad pressure to the disc equaling N = 25 kN and the brake mass being M = 5.7 t.

Based on the changes shown in Figure 7, it should be stated that with the change in the thickness of the friction linings (with the increase in the wear of the friction linings), the value of the average coefficient of friction decreases. This relationship was observed for all the analyzed braking speeds (from 50 to 200 km/h).

## 4. Regression Model of Friction Lining Wear and *μ_m_* Coefficient Variability Based on the Analysis of Vibration Acceleration Signals in the Amplitude Domain

**Figure 8:**
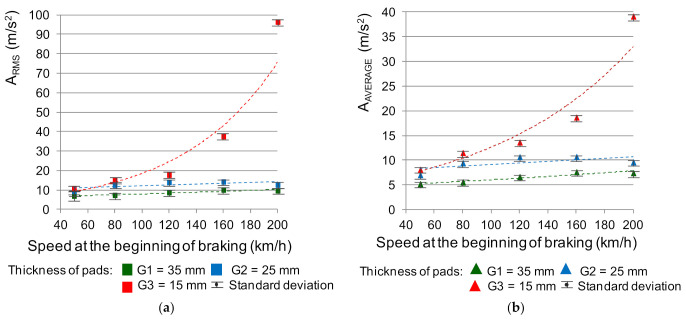
Dependence of the measure of vibration acceleration on the speed at the beginning of braking and the wear of the friction linings: (**a**) A_RMS_ effective value, (**b**) average value A_Average_.

The literature contains the results of research [39,40] on the use of vibroacoustics to diagnose the wear of brake friction linings in both road and rail vehicles. Then, such measures as the effective value of A_RMS_ vibration accelerations described by the relationship (3), or the average value A_Average_ (relationship (4)), are used. In the diagnostics of the braking system, the above-mentioned measures have the highest value of the dynamics of changes in the diagnostic parameter. However, in the scope of modeling the value of the average coefficient of friction of a disc brake, it should first be shown that there is a dependence of changes in the value of the vibration signal on the condition of the friction linings (their wear). Then, it would be possible to use these relationships when modeling the friction coefficient, which strongly depends on the wear of the friction material [41,42]. Figure 8 shows the dependence of the effective and mean value of vibration accelerations on braking at speeds of 50, 80, 120, 160 and 200 km/h. The results of the VA tests were adjusted in the form of the effective value and the mean value of vibration accelerations for the analyzed speeds using the least squares method. On this basis, regression models with a visible coefficient of determination R^2^ were determined. Each point on the graph in Figure 8 is the average of eight measurements. The number of repetitions was based on previous tests carried out on a 40-braking test. These vibroacoustic tests were carried out on the vibrations recorded on the linings on the right and left side of the brake disc. During the preliminary tests, the mean value and variance (standard deviation) were calculated for each successive braking. Then, the coefficient of variation was calculated. It has been observed that the value of the coefficient of variation is below 10% with the braking. Then, the stationarity and ergodicity of the recorded vibration acceleration signals were checked. The study of the nature of the signals influenced the establishment of the later methodology of the main research.

A diagnostic regression model was used in the analysis of vibroacoustic signals. This model assumes that the condition parameters (lining wear) influence the signal parameters. One-dimensional linear models and one-dimensional non-linear models were used. The choice of the regression model depended on the obtained coefficient of determination R^2^. For the test points, the fit was checked with a linear function and various non-linear functions. The selection of the function depended on the highest value of the R^2^ determination coefficient. Changes in the values of measures (A_RMS_ and A_AVERAGE_) as a function of speed at the beginning of braking for different friction lining thicknesses were described by the following linear and non-linear functions:

(7) $$A_{\mathrm{RMS}}=0.0219\times v_{0}\;+\;5.8382;\;\;\mathrm{for}\;G_{1}=35\;\mathrm{mm}$$

(8) $$A_{\mathrm{RMS}}=0.0202\times v_{0}\;+\;10.034;\;\;\mathrm{for}\;G_{2}=25\;\mathrm{mm}$$

(9) $$A_{\mathrm{RMS}}=4.517\;e^{0.0141\;v_{0}};\;\;\mathrm{for}\;G_{3}=15\;\mathrm{mm}$$

(10) $$A_{\mathrm{AVERAGE}}=0.017\times v_{0}\;+\;4.3879;\;\;\mathrm{for}\;G_{1}=35\;\mathrm{mm}$$

(11) $$A_{\mathrm{AVERAGE}}=0.0162\times v_{0}\;+\;7.4859;\;\;\mathrm{for}\;G_{2}=25\;\mathrm{mm}$$

(12) $$A_{\mathrm{AVERAGE}}=4.8282\;e^{0.0096\;v_{0}};\;\;\mathrm{for}\;G_{3}=15\;\mathrm{mm}$$

Table 1 shows the values of measures determined from the braking process for different speeds at the beginning of braking along with the dynamics of changes.

**Figure 9:**
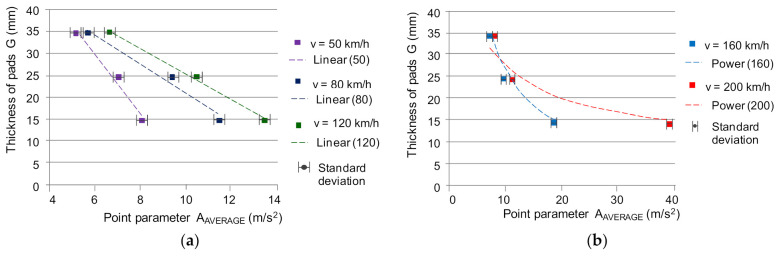
Dependence of the friction lining thickness on the effective value of vibration acceleration for the braking speed: (**a**) *v* = 50, 80 and 120 km/h, (**b**) *v* = 160 and 200 km/h.

The analysis of the test results showed that there is a dependence of the measurement values on the wear of the friction linings by measuring the vibrations on the brake holder. Only for low speeds (up to 50 km/h), the dynamics of changes of the most worn lining up to a thickness of 15 mm does not reach 6 dB. For the remaining speeds at the beginning of braking 80–200 km/h, the dynamics of changes is 6–19 dB. Due to the dynamic nature of the braking process, especially at high speeds, vibrations generated by the brake system provide information about the wear of the friction elements. On the other hand, the dependence of the friction coefficient on the speed and condition of the friction material will make it possible to evaluate the braking process (the value of the average coefficient of friction). To assess the wear of the friction linings, the inverse function to the approximating functions described by the relations (7)–(12) should be used; allowing estimation of the thickness of the friction linings on the basis of the measurement values. Figure 9 presents the dependence of the friction lining thickness on the average A_AVERAGE_ value. In the case of the RMS effective value, similar characteristics were obtained.

The dependence of the friction lining thickness on the value of the measures due to the highest value of the coefficient of determination R^2^ was approximated by the following linear functions for the beginning of braking at speeds from 50 to 120 km/h, and power functions for speeds of 160 and 200 km/h:

(13) $$G=-6.7505\times A_{\mathrm{AVERAGE}}+70.583;\;\;\mathrm{for}\;v=50\;\mathrm{km}/h$$

(14) $$G=-3.2772\times A_{\mathrm{AVERAGE}}+53.854;\;\;\mathrm{for}\;v=80\;\mathrm{km}/h$$

(15) $$G=-2.8412\times A_{\mathrm{AVERAGE}}+54.203;\;\;\mathrm{for}\;v=120\;\mathrm{km}/h$$

(16) $$G=233.72\times A_{\mathrm{AVERAGE}}{}^{-\;0.94};\;\;\mathrm{for}\;v=160\;\mathrm{km}/h$$

(17) $$G=79.597\times A_{\mathrm{AVERAGE}}{}^{-\;0.46};\;\;\mathrm{for}\;v=200\;\mathrm{km}/h$$

**Figure 10:**
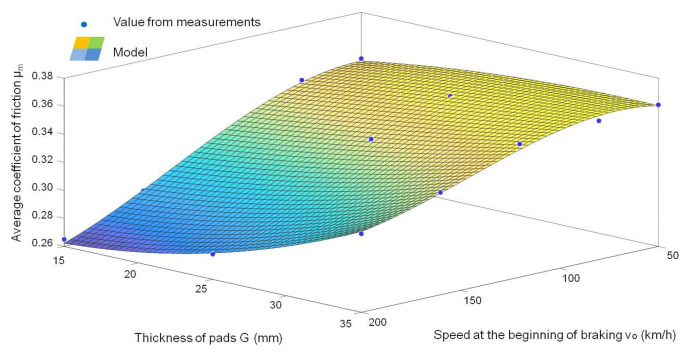
Dependence of the average coefficient of friction μ_m_ on the braking speed for N = 25 kN, M = 5.7 t and three friction lining thicknesses.

Based on the dependence (6) of the value of the average coefficient of friction obtained from the tests, the characteristics of changes in the average friction coefficient as a function of speed and wear of the friction material were determined (Figure 10).

It can be concluded that the value of the average coefficient of friction decreases with the increase in wear of the friction linings (reduction of their thickness) and the increase in speed at the beginning of braking. The regression model of the value of the average coefficient of friction as a function of the speed at the beginning of braking and the thickness of the friction linings shows the relationship:

(18) $$\mu_{m}=\lambda_{1}v_{0}+\lambda_{2}G+\lambda_{3}\upsilon_{0}^{2}+\lambda_{4}\nu_{0}G+\lambda_{5}G^{2}+\lambda_{6}\nu_{0}^{3}+\lambda_{7}G\nu_{0}^{2}+\lambda_{8}\nu_{0}G^{2}+\lambda_{0}$$

The final form of the regression model of the average coefficient of friction, depending on *v_o_* and *G* after verification of the parameters of the multiple regression model, is represented by the relationship:

(19) $$\mu_{m}=\lambda_{1}v_{0}+\lambda_{2}G+\lambda_{3}\upsilon_{0}^{2}+\lambda_{4}\nu_{0}G+\lambda_{5}G^{2}+\lambda_{6}\nu_{0}^{3}+\lambda_{7}G\nu_{0}^{2}+\lambda_{8}\nu_{0}G^{2}$$

Table 2 presents the values of the multiple regression function coefficients with the coefficient of determination R^2^ for the *μ_m_* model before and after the verification of the model parameters.

**Figure 11:**
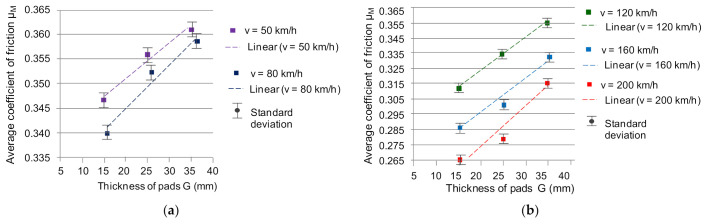
Dependence of the average coefficient of friction *μ_m_* on the thickness of the friction linings for the speed at the beginning of braking: (**a**) *v* = 50 and 80 km/h, (**b**) *v* = 120, 160 and 200 km/h.

The regression model of the variability of the average friction coefficient (after verification of the model coefficients), described with the relationship (19), is based on two variables: the speed of the beginning of braking and the thickness of the friction linings. However, to estimate the value of the average coefficient of friction taking into account the vibrations of the friction linings, a different model was used (two functions model). The relationship between the thickness of the friction linings and the average A_AVERAGE_ value of the acceleration of the linings vibration and the dependence of *μ_m_* on the thickness of the friction linings was used. The dependence of *μ_m_* on the thickness of the friction linings was presented in Figure 11.

The average coefficient of friction was approximated by a linear function, which is represented by the following relationships:

(20) $$\mu_{m}=7\times 10^{–4}G+0.34;\;\;\mathrm{for}\;v=50\;\mathrm{km}/h$$

(21) $$\mu_{m}=9\times 10^{–4}G+0.33;\;\;\mathrm{for}\;v=80\;\mathrm{km}/h$$

(22) $$\mu_{m}=21\times 10^{–4}G+0.28;\;\;\mathrm{for}\;v=120\;\mathrm{km}/h$$

(23) $$\mu_{m}=23\times 10^{–4}G+0.25;\;\;\mathrm{for}\;v=160\;\mathrm{km}/h$$

(24) $$\mu_{m}=26\times 10^{–4}G+0.22;\;\;\mathrm{for}\;v=200\;\mathrm{km}/h$$

Due to the existence of a linear dependence (at low speeds during braking) or non-linear (at high speeds during braking) between the thickness of the friction linings and the values of the measures, as well as the linear relationship for all speeds during braking of the average coefficient of friction on the lining thickness, the dependence was determined by the method of substituting two functions of the average coefficient of friction on the basis of registered vibrations. The general form of estimating the average value of the friction coefficient is presented in Equations (25) and (26). In the A_RMS_ example, Equation (25) is calculated based on two linear functions, and Equation (26) is the result of combining a non-linear function with a linear function.

(25) $$\left\{\begin{array}{c} {G=a}_{1}\times A_{\mathrm{RMS}}+\;b_{1}\\ \mu_{m}{=a}_{2}\;\times \;G\;{+b}_{2} \end{array}\right.$$

$$\mu_{m}{=a}_{1}\times a_{2}\;\times \;A_{\mathrm{RMS}}\;+\;a_{2}\times b_{1}\;+\;b_{2}$$

$$\mathrm{for}\;v_{0}\;=\;50,\;80,\;120\;\left[\frac{\mathrm{km}}{h}\right]$$

(26) $$\left\{\begin{array}{c} {G=c}_{1}\times A_{\mathrm{RMS}}{}^{d_{1}}\\ \mu_{m}{=c}_{2}\;\times \;G\;{+d}_{2} \end{array}\right.$$

$$\mu_{m}{=c}_{1}\times c_{2}\;\times \;A_{\mathrm{RMS}}{}^{d_{1}}\;+\;d_{2}\;\mathrm{for}\;v_{0}\;=\;160,\;200\;\left[\frac{\mathrm{km}}{h}\right]$$

where:

*a*_1_—multiplier of the linear model of the friction lining thickness as a function of A_RMS_ or A_AVERAGE_ for *v_0_* = 50, 80 and 120 km/h;
*b*_1_—free term of the linear model of friction lining thickness in the A_RMS_ or A_AVERAGE_ function for *v_0_* = 50, 80 and 120 km/h;
*c*_1_—directional coefficient of the non-linear (power) model of the friction lining thickness as a function of A_RMS_ or A_AVERAGE_ for *v_0_* = 160 and 200 km/h;
*d*_1_—exponent of the non-linear model of friction lining thickness in the A_RMS_ or A_AVERAGE_ function for *v_0_* = 160 and 200 km/h;
*a*_2_—multiplier of the linear model of the average friction coefficient as a function of the friction lining thickness for *v_0_* = 50, 80 and 120 km/h;
*b*_2_—free term of the linear model of the average friction coefficient as a function of the friction lining thickness for *v_0_* = 50, 80 and 120 km/h;
*c*_2_—multiplier of the linear model of the average friction coefficient as a function of the friction lining thickness for *v_0_* = 160 and 200 km/h;
*d*_2_—free term of the linear model of the average friction coefficient as a function of the friction lining thickness for *v_0_* = 160 and 200 km/h.

Based on the dependence on the wear of the friction linings as a function of the value of the vibration acceleration measures and the average coefficient of friction on the wear of the friction linings, as well as the Functions (25) and (26), the following relationships were determined to estimate the average value of the friction coefficient:

(27) $$\mu_{m}=-0.0047\times A_{\mathrm{AVERAGE}}+0.386;\;\;\mathrm{for}\;v=50\;\mathrm{km}/h$$

(28) $$\mu_{m}=-0.0029\times A_{\mathrm{AVERAGE}}+0.376;\;\;\mathrm{for}\;v=80\;\mathrm{km}/h$$

(29) $$\mu_{m}=-0.0059\times A_{\mathrm{AVERAGE}}+0.394;\;\;\mathrm{for}\;v=120\;\mathrm{km}/h$$

(30) $$\mu_{m}=0.56\times A_{\mathrm{AVERAGE}}{}^{-\;0.94}+0.249;\;\;\mathrm{for}\;v=160\;\mathrm{km}/h$$

(31) $$\mu_{m}=0.21\times A_{\mathrm{AVERAGE}}{}^{-\;0.46}+0.222;\;\;\mathrm{for}\;v=200\;\mathrm{km}/h$$

The results of the research showed that on the basis of the braking system vibration acceleration measures analyzed in this study, it is possible to both diagnose the condition of the brake, already described in [43], as well as to assess the braking process by determining the average coefficient of friction [44].

The relative percentage error in the representation of the regression model of the average coefficient of friction on the basis of the recorded signals of vibration accelerations during braking is approximately 1% at *v* = 50, 80 and 120 km/h. It occurs both with averaging the vibration acceleration signal with the A_RMS_ effective value and with the A_AVERAGE_ value. During braking from *v* = 160 km/h, the error in estimating the average value of the coefficient of friction is about 4% for A_RMS_ and 2.5% for A_AVERAGE_. Braking at *v* = 200 km/h causes an error in the estimation of 6% for both the average and the effective value.

**Figure 12:**
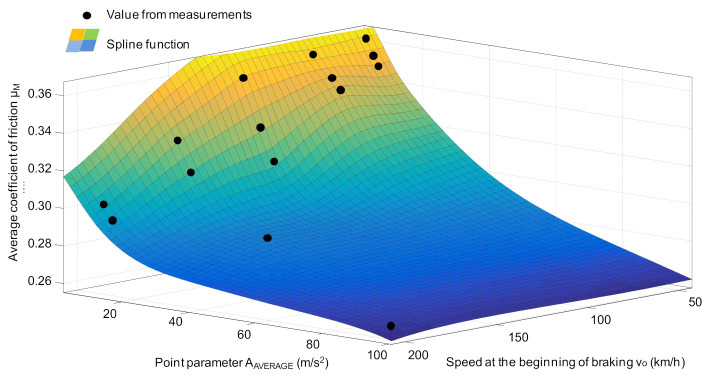
Dependence of the average coefficient of friction μ_m_ on the A_AVERAGE_ function and velocity *v*_0_, obtained from the tests in relation to the regression model obtained from Equations (27)–(31) on the example of the spline function.

Figure 12 presents a graphic representation of the match of the regression model for estimating the value of the average coefficient of friction on the basis of the average value of the vibration acceleration of the friction linings.

**Figure 13:**
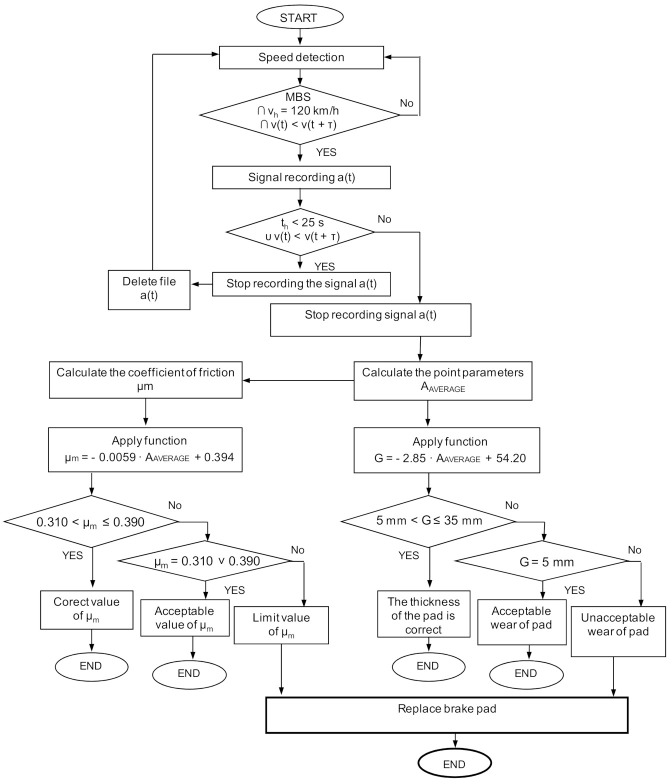
Algorithm of wear evaluation and estimation of the average coefficient of friction during braking at *v* = 120 km/h; MBS—perform service stopping braking, τ—increase of the braking time.

Based on the relationships (13)–(17) and (27)–(31), on the example of the average value of vibration accelerations, it is possible to develop a diagnostic algorithm for the simultaneous assessment of the brake friction material wear and determination of the average friction coefficient for the railway disc brake. For the selected speed at the beginning of braking, Figure 13 shows an algorithm which, in the three-state evaluation (serviceability, acceptability and limit condition), determines the wear range of the friction linings and the value of the average coefficient of friction.

The algorithm uses data from the measurement of vehicle speed and vibrations of the braking system, functions describing wear and the coefficient of friction. The wear assessment is performed in three ranges, i.e., for the thickness of the friction linings from 5 to 35 mm, for the thickness of 5 mm, and less than 5 mm. For the average coefficient of friction, its value is estimated from 0.310 to 0.390, as recommended in accordance with the UIC 541-3 sheet. Values equal to 0.390 and 0.310 are considered acceptable, while values lower than 0.310 are the limit value of this parameter. When the limit values for both the friction lining wear and the friction coefficient are reached, a message appears to the driver to send the vehicle for inspection.

## 5. Regression Model of Friction Lining Wear and *μ_m_* Variability Based on the Analysis of Vibration Acceleration Signals in the Frequency Domain

**Figure 14:**
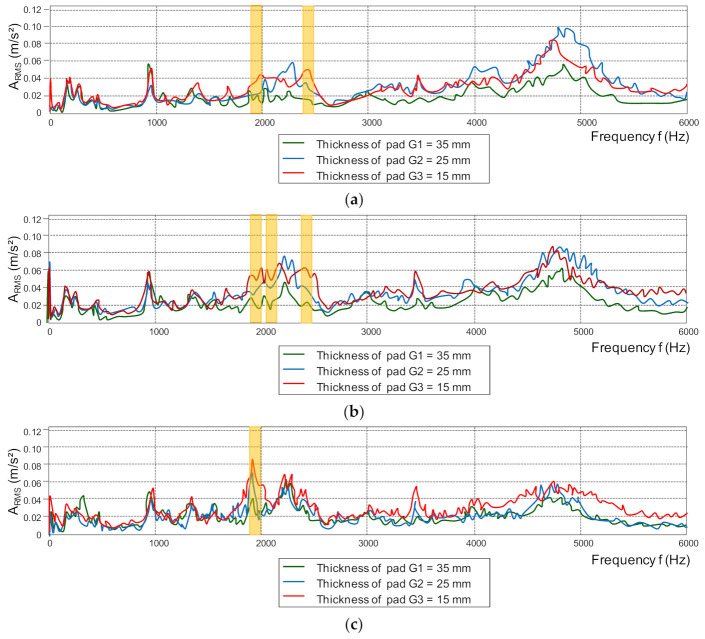
Dependence of the amplitude of vibration accelerations on the frequency for different friction lining thicknesses when braking from the speed: (**a**) *v* = 120 km/h, (**b**) *v* = 160 km/h, (**c**) *v* = 200 km/h.

Spectral analysis, allow identification of the wear of the friction linings with greater accuracy (higher value of the dynamics of changes than in the analysis in the amplitudes domain). Due to the dependence of the change in the brake friction coefficient on the wear of the friction material (Figure 10), the *µ_m_* value on the basis of the vibrations generated by the friction linings during braking was estimated. The main purpose of the spectral analysis of vibration signals was to determine common frequency bands related to the change in the lining thickness during the operation of the brake system in a wide braking range (speed from 50 to 200 km/h). Then, from a given frequency band, the effective value of vibration accelerations was determined in accordance with the relationship (4), and the parameter of dynamics of changes in dB was checked, in accordance with the relationship (5). Figure 14 shows examples of amplitude spectra of vibration acceleration signals generated during braking by the friction linings.

The analysis of vibration acceleration signals in the spectral domain generated by the friction linings does not allow the finding of a common frequency band for all analyzed speeds and thicknesses of the friction linings. It is especially difficult when braking from low speeds (50 and 80 km/h). At these speeds, no increase in the RMS value from a given frequency band is observed with the wear of the brake components. For medium and high speeds during braking (120, 160 and 200 km/h), it is possible to determine the frequency bands in which the A_RMS_ changes depend on the friction lining wear (Table 3).

**Figure 15:**
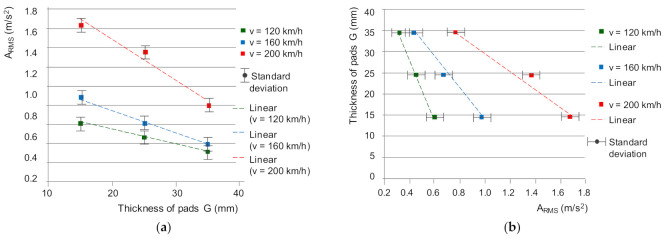
Characteristics of changes: (**a**) A_RMS_ = f (G), (**b**) G = f (A_RMS_) for three braking speeds in the 1950–2000 Hz frequency band.

For the common frequency band of 1950–2000 Hz, the vibration acceleration of the friction linings, taking into account both the three braking speeds (120, 160 and 200 km/h) and the wear of the friction linings, the A_RMS_ exchange characteristics were determined (Figure 15).

For diagnostic purposes, the dependence of the friction lining thickness on the effective value of vibration acceleration was determined (Figure 15b). The dependence was approximated by a linear function in order to derive the following linear regression models of friction lining wear assessment:

(32) $$G=-72.7\times A_{\mathrm{RMS}}+59.1;\;\;\mathrm{for}v=\;120\;\mathrm{km}/h$$

(33) $$G=-40.4\;\times A_{\mathrm{RMS}}+50.2;\;\;\mathrm{for}v=\;160\;\mathrm{km}/h$$

(34) $$G=-22.9\;\times A_{\mathrm{RMS}}+53.9;\;\;\mathrm{for}v=\;200\;\mathrm{km}/h$$

Then, regressive linear models of changes in the average coefficient of friction as a function of the thickness of the friction material presented in Figure 11b and described with the dependencies (22)–(24) were used. By substituting two linear functions G = f(A_RMS_) and *μ_m_* = f(G), in accordance with the general Equation (25), linear regression models of the average friction coefficient were determined based on the vibrations of the friction linings subjected to spectral analysis in the 1950–2000 Hz frequency band.

(35) $$\mu_{m}=\;-0.153\times A_{\mathrm{RMS}}+\;0.404;\;\;\mathrm{for}v=\;120\;\mathrm{km}/h$$

(36) $$\mu_{m}=\;-0.097\times A_{\mathrm{RMS}}+\;0.369;\;\;\mathrm{for}v=\;160\;\mathrm{km}/h$$

(37) $$\mu_{m}=\;-0.153\times A_{\mathrm{RMS}}+\;0.404;\;\;\mathrm{for}v\;=\;120\;\mathrm{km}/h$$

**Figure 16:**
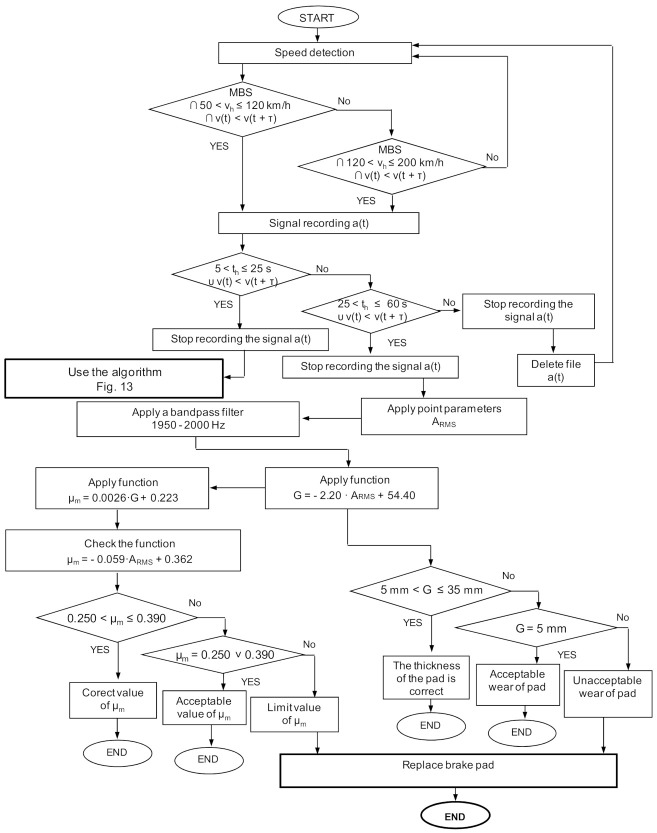
Algorithm of wear assessment and estimation of the average coefficient of friction during braking, for the speed *v* = 200 km/h using the spectral analysis; MBS—perform service stopping braking, τ—increase of the braking time.

The relative percentage error in the representation of the regression model of the average coefficient of friction on the basis of the recorded signals of vibration acceleration during braking does not exceed 3% for the analyzed speeds at the beginning of braking (*v* = 120, 160 and 200 km/h). It should be emphasized that the use of the spectral analysis with subsequent band filtering means that the diagnostic models in the assessment of friction lining wear and estimating the friction coefficient values are twice as accurate in relation to the analysis in the amplitudes domain. A significant drawback of spectral analysis is the difficulty of identifying a common frequency band for a wide range of braking speeds. The use of a common bandpass filter is possible only at medium and high speeds, while at low speeds (*v* = 50 and 80 km/h) the effective values of vibration acceleration are comparable with the values derived from the amplitude analysis. Figure 16 shows the algorithm (for a selected speed (200 km/h) to assess the wear of friction linings and to evaluate the braking process.

In the first stage of the algorithm, the analysis of vibration acceleration signals for low and medium speeds during braking is carried out, using measures and spectral analysis at speeds above 120 km/h. The algorithm presented in Figure 16 is a procedure that allows the determination of both the thickness of the friction linings and the average coefficient of friction on the basis of the spectral analysis. The value of the thickness of the friction linings and the value of the average coefficient of friction with the imposed tolerance of the average coefficient of friction are compliant with the UIC 541-3 sheet. For the algorithm presented in Figure 13, based on the analysis in the amplitudes domain, greater accuracy was obtained in mapping the regression models.

The tests were carried out on a certified stand for testing railway disc brakes in the 1:1 scale. Considering the fact that research on real objects was carried out, more attention was paid to the results of vibroacoustic tests, which can be directly used in railways. Hence, the obtained results were assessed quantitatively. Algorithms for assessing the condition of friction linings were proposed, i.e., for the assessment of wear and for the assessment of the braking process. The developed test methodology with vibration transducers can be directly applied in a real object, i.e., on a disc brake of a passenger car.

## 6. Conclusions

Based on the test results, it is possible to use the vibroacoustic diagnostics (WA) more widely and the following conclusions can be drawn:

- Besides the assessment of the technical object condition, the changes in the average coefficient of friction as a function of the braking speed can be determined. This is due to the strong dependence of the diagnostic parameter on wear expressed by the dynamics of changes, exceeding 6 dB, as well as the dependence of the average coefficient of friction on the speed and friction linings wear. The combination of both functions enables the determination of linear (at low speeds) and non-linear (at higher braking speeds) regression models to estimate the value of the average coefficient of friction;
- The error in matching the regression model of the average coefficient of friction on the basis of the determined measures during braking only at some speeds of the beginning of braking reaches the value of 6%;
- It is not possible to find a common frequency band for a wide range of braking speeds (from 50 to 200 km/h). The common frequency band in the range of 1950–2000 Hz enables the determination of the A_RMS_ dependence from the band for three cases of the friction material condition, and for medium and high speed when braking with the dynamics of changes exceeding 6 dB;
- The error of adjusting the results of operational tests to the values determined from the regression linear models of the coefficient of friction, determined with the relative percentage error, does not exceed 1% for *v* = 120 km/h, 3% for *v* = 160 km/h, and 1.5% for *v* = 200 km/h;
- For amplitude analyses, the error of fitting the regression model to the test results exceeds 6% for *v* = 200 km/h, 2.4–3.7% for *v* = 160 km/h, depending on the measure used, and about 1–1.5% for the average (120 km/h) and low braking speeds (50–80 km/h).

It should be emphasized that the use of vibration diagnostics to assess the wear of the friction material on the basis of amplitude analysis (for low speeds at the beginning of braking) and spectral analysis (for high speeds at the beginning of braking) additionally enables the assessment of the braking process in terms of changes in the average friction coefficient. Due to the imposed wear limits of the friction material and the tolerance of the average coefficient of friction, it is possible to apply simple algorithms based on measuring the speed of the rail vehicle and vibrations from the braking system. Then, after reaching the limit wear of the friction linings (below 5 mm) or the value of *μ_m_* exceeding the lower or upper tolerance, as a result of, i.e., damage or burning of the friction surface of the lining, a message appears on the control panel that the linings need to be replaced without visual inspection of the brake system.

## Figures and Tables

**Table 1:** The values of the measures of the vibration acceleration of the friction linings depending on the speed of braking and the wear of the friction linings, along with the dynamics of changes.

**Measure**	**Values of Measure m/s** ^**2**^			**Dynamics of Change dB**
	**Cladding Thickness G**_**1**_ ** = 35 mm**	**Cladding Thickness G**_**2**_ ** = 25 mm**	**Cladding Thickness G**_**3**_ ** = 15 mm**	
Braking start at *v*_0_ = 50 km/h				
A_RMS_	6.88	9.36	10.63	3.78
A_AVERAGE_	5.18	7.01	8.07	3.86
Braking start at *v*_0_ = 80 km/h				
A_RMS_	7.29	12.47	15.05	6.29
A_AVERAGE_	5.55	9.39	11.48	6.32
Braking start at *v*_0_ = 120 km/h				
A_RMS_	8.65	13.95	17.68	6.21
A_AVERAGE_	6.61	10.62	13.60	6.26
Braking start at *v*_0_ = 160 km/h				
A_RMS_	10.03	14.17	37.46	11.4
A_AVERAGE_	7.59	10.67	18.66	7.8
Braking start at *v*_0_ = 200 km/h				
A_RMS_	9.68	12.57	96.31	19.7
A_AVERAGE_	7.40	9.59	39.12	14.5

**Table 2:** Regression function coefficients with R^2^ fitting for the *μ_m_* model.

**Before Verification**			**After Verification**	
**Coefficient**	**Coefficient Value**	Value F *	**Coefficient Value**	Value F *
λ_1_	0.00093858	0.01225	0.001393	0.001667
λ_2_	0.0043017	0.004541	0.005225	0.001726
λ_3_	–1.035 × 10^−5^	0.00921	–1.2173 × 10^−5^	0.005887
λ_4_	–2.202 × 10^−5^	0.06318	–4.0207 × 10^−5^	0.004797
λ_5_	–0.0001027	0.005457	–0.0001027	0.002679
λ_6_	2.778 × 10^−8^	0.009771	2.7788 × 10^−8^	0.01291
λ_7_	–7.283 × 10^−8^	0.08624	1.0718 × 10^−6^	0.001060
λ_8_	1.072 × 10^−6^	0.001620		
λ_0_	0.2885	4.9607 × 10^−24^	0.2654	3.3132 × 10^−26^
R^2^	0.996	1.04 × 10^−7^ *	0.994	2.52 × 10^−8^ *

* significance for a particular regression coefficient.

**Table 3:** A_RMS_ values from the frequency bands of the vibration acceleration of the friction linings depending on the speed of braking and the wear of the friction linings along with the dynamics of changes.

**Frequency Hz**	A_**RMS**_** Value m/s**^**2**^			**Dynamics of Change dB**
	**Cladding Thickness G**_**1**_ ** = 35 mm**	**Cladding Thickness G**_**2**_ ** = 25 mm**	**Cladding Thickness G**_**3**_ ** = 15 mm**	
Braking start at *v* = 120 km/h				
1950–2000	0.33	0.46	0.61	5.20
2450–2500	0.29	0.48	0.64	7.00
Braking start at *v* = 160 km/h				
1950–2000	0.39	0.59	0.88	7.08
2050–2100	0.46	0.75	0.89	5.81
2450–2500	0.38	0.54	0.99	8.34
Braking start at *v* = 200 km/h				
1950–2000	0.78	1.35	1.64	6.27
3400–3450	0.38	0.66	0.98	8.19
5050–5100	0.49	0.63	1.02	6.41
5300–5350	0.35	0.39	0.75	6.65

## Data Availability

The data presented in this study are available on request from the corresponding author.
